# Development and validation of a prediction model for in-hospital mortality among patients with acute diquat poisoning: A retrospective cohort study

**DOI:** 10.1097/MD.0000000000045613

**Published:** 2025-11-14

**Authors:** Ye Zhang, Xian Chen, Min Zhao, Haike Du, Xiaoming Jiang, Yingmin Ma

**Affiliations:** a Department of Respiratory and Critical Care Medicine, Beijing You’an Hospital, Capital Medical University, Beijing, PR China; b Department of Critical Care Medicine, Beijing Mentougou District Hospital, Beijing, PR China; c Department of Critical Care Medicine, Beijing Tongren Hospital Mentougou Campus, Capital Medical University, Beijing, PR China; d Department of Critical Care Medicine, Capital Medical University Mentougou Teaching Hospital, Beijing, PR China; e Department of Emergency, Chinese People’s Armed Police Force Characteristic Medical Center, Tianjing, PR China; f Department of Emergency, Xinqiao Hospital, Army Medical University, Chongqing, PR China.

**Keywords:** acute diquat poisoning, in-hospital mortality, nomogram, prediction, prognosis

## Abstract

Diquat has become the leading bipyridine herbicide, surpassing paraquat and causing more poisoning cases, yet knowledge about it is limited. Our study sought to create and validate a nomogram to predict in-hospital mortality in patients with acute diquat poisoning. This retrospective cohort study, conducted from January 2016 to April 2024 in a tertiary hospital, identified prognostic factors using best subsets regression with the lowest Bayesian information criterion. A nomogram based on 5 prognostic factors was validated through bootstrap sampling validation and leave-one-out cross-validation. The model’s effectiveness was assessed using a decision analysis curve. Of the 98 acute acute diquat poisoning cases included, 58.2% were male, with a 34.7% in-hospital mortality rate. Five prognostic factors (post-ingestion time, plasma diquat concentration, plasma lactate concentration, occurrence of respiratory failure and need for blood transfusion) were used to create a predictive nomogram with area under the curve was 0.874 (95% confidence interval: 0.806–0.941) and concordance index of 0.874. For the bootstrap sampling validation, the corrected concordance index was 0.846. In the leave-one-out cross-validation, the concordance index was 0.849. The Hosmer–Lemeshow test (χ^2^ = 8.125, *P* = .421) and brier score (original 0.142, mean 0.167 in cross-validation) supported this. Clinical decision analysis indicated patient benefit at threshold probabilities of 1% to 76%. We developed a predictive model and nomogram to forecast in-hospital mortality in acute diquat poisoning patients using baseline characteristics. This model performs well and can help clinicians identify patients at high risk of in-hospital mortality.

## 1. Introduction

Paraquat and diquat are nonselective contact herbicides that effectively control the growth of weeds and grasses, thereby enhancing agricultural productivity.^[1–4]^ Presently, paraquat has been discontinued in many countries due to its high toxicity,^[5–8]^ and diquat was used as its substitute. Consequently, there has been a rise in the incidence of acute diquat poisoning in recent years.^[9–12]^

There is no specific antidote for acute diquat poisoning.^[13]^ It is typically characterized by initial symptoms such as kidney and liver function impairment and, in severe cases, can result in multiple organ failure and death.^[7,12–14]^ Currently, relevant literature reports that the in-hospital mortality rate of patients with acute diquat poisoning exceeds 20%, imposing a relatively heavy burden on patients and the healthcare system.^[15–18]^ Therefore, gaining a comprehensive understanding of mortality among patients with acute diquat poisoning and its associated factors is essential for the prevention and management of this condition.

Many risk factors associated with prognosis of acute diquat poisoning have been reported, including plasma diquat concentration, plasma lactate concentration, white blood count, aspartate aminotransferase, alanine aminotransferase and serum creatinine.^[18,19]^ Our previous studies had also confirmed that the initial plasma diquat concentration, as well as the severity-index and toxicity-index of diquat calculated from the plasma diquat concentration and poisoning time were associated with patient outcomes.^[20,21]^ However, few studies have been conducted to develop and validate a nomogram to estimate the in-hospital mortality among patients with acute diquat poisoning. Our goal is to use clinically available data to develop a predictive model that can help clinicians identify early patients with acute diquat poisoning who have a poor prognosis.

## 2. Materials and methods

### 2.1. Study setting and patients

This was a retrospective cohort study involving patients diagnosed with acute diquat poisoning from January 2016 to April 2024 at the Chinese People’s Armed Police Force Characteristic Medical Center. This is a tertiary hospital with 1900 beds, an annual emergency department visit volume of 1,00,000, and a population coverage of 1 million.

#### 2.1.1. Inclusion criteria

Patients who had been admitted to the hospital within 24 hours of ingesting diquat should be confirmed through plasma diquat concentrations.

#### 2.1.2. Exclusion criteria

Patients under 14 years of age; patients with incomplete data records; patients died or discharged within 24 hours; patients with concurrent drug, paraquat, other herbicide, natural toxin, or chemical poisoning; patients with underlying diseases such as chronic obstructive pulmonary disease, heart failure, chronic kidney disease, or chronic liver disease. Importantly, considering the possibility of the presence of paraquat in the herbicide, all patients underwent laboratory testing to exclude the possibility of co-poisoning with paraquat or other pesticides.

### 2.2. Study procedure and measurements

Data were gathered from medical records for the study period. For those with a history of ingesting diquat and showing relevant symptoms, their initial plasma diquat concentration was measured immediately upon hospital arrival. Post-ingestion time was defined as the time from diquat ingestion to measurement. Upon emergency department arrival, blood samples were taken for measurements of plasma diquat concentration, liver function tests, renal function tests, cardiac enzymes and electrocardiograph for cardiac injuries, plasma lactate concentration, complete blood counts.

Complications like seizures, kidney or liver insufficiencies, myocardial injury, gastrointestinal bleeding, respiratory failure, and coagulation issues, along with treatments like mechanical ventilation, need for blood transfusion, hemoperfusion, and continuous kidney replacement therapy were documented.

Initial plasma diquat concentration was measured using spectrophotometry. Spectrophotometry was used to quickly measure diquat by adding the sample to 1.5 mL of 1 mmol/L sodium hydroxide with 1% sodium dithionite. The green reduction product’s absorbance was read at 770 nm.

Acute diquat poisoning was treated by preventing absorption through gastric-lavage, adsorption, catharsis, and whole bowel irrigation.^[3,7,10,22,23]^ Lavage the stomach with plenty of normal saline or warm water via the gastric-lavage machine. Polyethylene glycol was employed for whole bowel irrigation if necessary. Diquat elimination was facilitated by diuretics, hemoperfusion, or continuous renal replacement therapy. Mannitol prevented cerebral edema, corticosteroids reduced inflammation, vitamin C served as an antioxidant, and oxygen therapy or mechanical ventilation addressed hypoxia or respiratory failure.

The outcome (in-hospital mortality) strictly encompassed deaths occurring before the completion of discharge procedures within our hospital system. This included deaths in any clinical unit (e.g., emergency department, intensive care unit, general wards) during a continuous hospitalization episode without transfer-out events. All cases were tracked via the institution-wide electronic health record system, which automatically logs real time mortality data across all departments. No inter-hospital transfer cases were included in this study.

### 2.3. Ethics statement

The research was carried out following the guidelines of the Declaration of Helsinki and received approval from the Institutional Review Board of Beijing Mentougou District Hospital (Approval number MQLW230701). Due to the retrospective design of the study, the necessity for obtaining informed consent was waived by the Institutional Review Board.

### 2.4. Statistical analysis

Patients were divided into survival and non-survival groups based on outcomes. Categorical variables were shown as frequencies or percentages, and continuous variables as means and standard deviation for normal data, or median and interquartile range for skewed data. Statistical analyses included 1-way ANOVA for normal data, Kruskal–Wallis *H* test for skewed data, and chi-square tests for categorical variables to evaluate group differences.

Imputation was applied to variables with <20% missing data. Variables with >20% missing data (e.g., procalcitonin, neutrophil gelatinase-associated lipocalin) were excluded due to insufficient availability for reliable imputation or analysis. Following the transparent reporting of a multivariable prediction model for individual prognosis or diagnosis guidelines,^[24]^ we developed and validated a monogram to predict in-hospital mortality by taking these steps. First, we used the minimum Bayesian information criterion (BIC) and best subsets regression to select potential prognostic factors. Second, these factors were used to build the model, which was then visualized with a monogram. Third, the prediction model’s discrimination and calibration were assessed using receiver operating characteristic curve (reported by area under the curve [AUC] and 95% confidence interval), concordance index, and calibration curve analyses (reported by brier score, and Hosmer–Lemeshow test), with validation made via 200 bootstrap samples. Additionally, leave-one-out cross-validation (internal cross-validation) was conducted to further evaluate model performance. Finally, a decision analysis curve was used to evaluate the application and impact of the model.

Analyses were conducted using R version 4.2.2 and Free Statistics software version 1.9, with 2-sided *P* values <.05 deemed significant.

## 3. Results

### 3.1. Baseline characteristics of subjects

**Figure 1.**
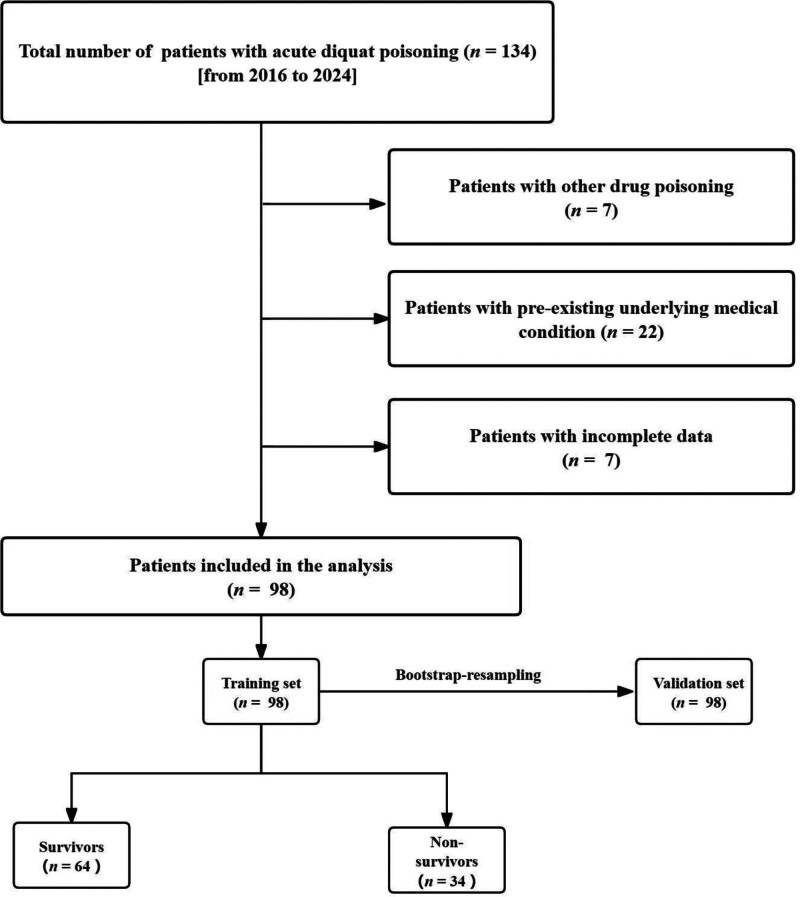
Flowchart of the study sample.

The study initially identified 134 patients through case retrieval, excluding 36 patients (including 7 with concurrent other drug poisoning, 22 with underlying diseases, and 7 with incomplete data). Ultimately, 98 cases of acute paraquat poisoning were included for analysis (Fig. 1). As shown in Table 1, there were 64 survivors and 34 non-survivors, with an in-hospital mortality rate of 34.7%, of which 58.2% were male. The age distribution was 33.3 ± 12.3 years, with a median post-ingestion time of 252.5 minutes, and the hospitalization duration ranged from 3 to 29 days.

**Table 1:** Baseline characteristics of the study participants.

Variables	Total (n = 98)
Prognosis, n (%)	
Survivors	64 (65.3)
Non-survivors	34 (34.7)
Sex, n (%)	
Male	57 (58.2)
Female	41 (41.8)
Age (yr), median (interquartile range)	33.3 ± 12.3
White blood cell count (×10^12^/L), mean ± standard deviation (reference value 3.5–9.5 × 10^9^/L)	20.6 ± 9.4
Alanine aminotransferase activity (U/L), median (interquartile range; reference value 0–35 U/L)	29.0 (23.0, 60.0)
Aspartate aminotransferase activity (U/L), median (interquartile range; reference value 14–36 U/L)	32.0 (19.2, 62.8)
Creatinine concentration (μmol/L; mg/dL), median (interquartile range; reference value 46–92 μmol/L [0.52–1.04 mg/dL])	75.0 (54.0, 132.8; 0.85 [0.61–1.50])
Lactate concentration (mmol/L), median (interquartile range; reference value 0.5–2.2 mmol/L)	2.5 (1.5, 4.8)
Post-ingestion time (min), median (interquartile range)	252.5 (190.0, 398.8)
Plasma diquat concentration (mg/L), median (interquartile range)	2.2 (1.6, 3.6)
Continuous kidney replacement therapy, n (%)	
No	29 (29.6)
Yes	69 (70.4)
Hemoperfusion, n (%)	
No	25 (25.5)
Yes	73 (74.5)
Blood transfusion, n (%)	
No	74 (75.5)
Yes	24 (24.5)
Ventilation, n (%)	
No	58 (59.2)
Yes	40 (40.8)
Seizure, n (%)	
No	70 (71.4)
Yes	28 (28.6)
Liver insufficiency, n (%)	
No	29 (29.6)
Yes	69 (70.4)
Kidney insufficiency, n (%)	
No	32 (32.7)
Yes	66 (67.3)
Respiratory failure, n (%)	
No	48 (49.0)
Yes	50 (51.0)
Gastrointestinal haemorrhage, n (%)	
No	46 (46.9)
Yes	52 (53.1)
Coagulation dysfunction, n (%)	
No	33 (33.7)
Yes	65 (66.3)
Pneumonia, n (%)	
No	39 (39.8)
Yes	59 (60.2)
Myocardial injury, n (%)	
No	49 (50.0)
Yes	49 (50.0)

### 3.2. Variables selection using the best subsets regression

**Figure 2.**
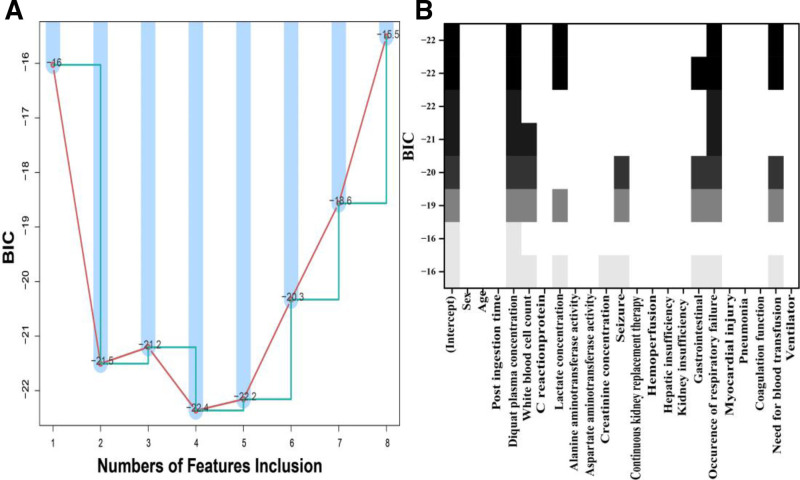
The selection of variables using the best subsets regression method. (A) All 22 candidate parameters evaluated by best-subsets regression; the combination with the lowest Bayesian information criterion (BIC = −22.4) is indicated. (B) Final parsimonious model retaining four core variables: plasma diquat concentration, plasma lactate concentration, respiratory failure and need for blood transfusion. BIC = Bayesian information criterion.

The best subsets regression method effectively selects variables by evaluating all combinations and choosing the set with the lowest BIC. Figure 2A identifies all 22 parameters with a minimum BIC of −22.4, while Figure 2B highlights the final model, which includes 4 key variables: plasma diquat concentration, plasma lactate concentration, occurrence of respiratory failure, and need for blood transfusion. This model incorporates clinical factors from the primary cohort. Simultaneously, prior research has demonstrated a significant correlation between the post-ingestion time and the prognosis of patients affected by acute diquat poisoning; therefore, post-ingestion time was also incorporated into this model.^[19–21]^ Thus, 5 prognostic factors were used to create the nomogram.

### 3.3. Prognostic nomogram

**Figure 3.**
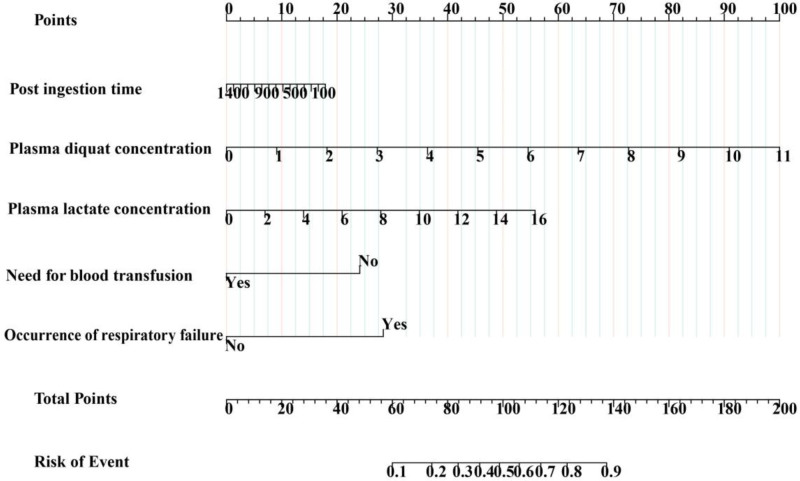
Nomogram to predict the probability of in-hospital mortality in patients with acute diquat poisoning. According to nomogram, points for diquat plasma concentration, plasma lactate concentration, occurrence of respiratory failure, need for blood transfusion and post-ingestion time can be calculated from first line. Total points were the sum of the 5 points. And we can evaluate the risk of in-hospital mortality from the last line according to the total point.

Figure 3 presents a nomogram for predicting in-hospital mortality in acute diquat poisoning patients. It assigns points based on post-ingestion time, plasma diquat concentration, plasma lactate concentration, occurrence of respiratory failure, and need for blood transfusion. The total score, derived from summing these points, indicates the mortality risk on the last line.

The specific calculation equation is as follows:


$$\begin{array}{l} \;\begin{array}{l} \begin{array}{lll} & Logistics\;\left(probability\;of\;in\text{-}hospital\;motality\right)\; & =-3.217-0.001\;\left(post\text{-}ingestion\;time\right)\;\; \end{array}\;\begin{array}{lll} & +0.515\left(plasma\;diquat\;concentration\right)\; & +0.198\;\;\left(plasma\;lactate\;concentration\right)\; \end{array}\;\begin{array}{lll} & +1.607\left(occurence\;of\;respiratory\;failure\right)\; & -1.366\;\left(need\;for\;blood\;transfusion\right).\; \end{array}\; \end{array} \end{array}$$

### 3.4. Discrimination and calibration of the nomogram

**Figure 4.**
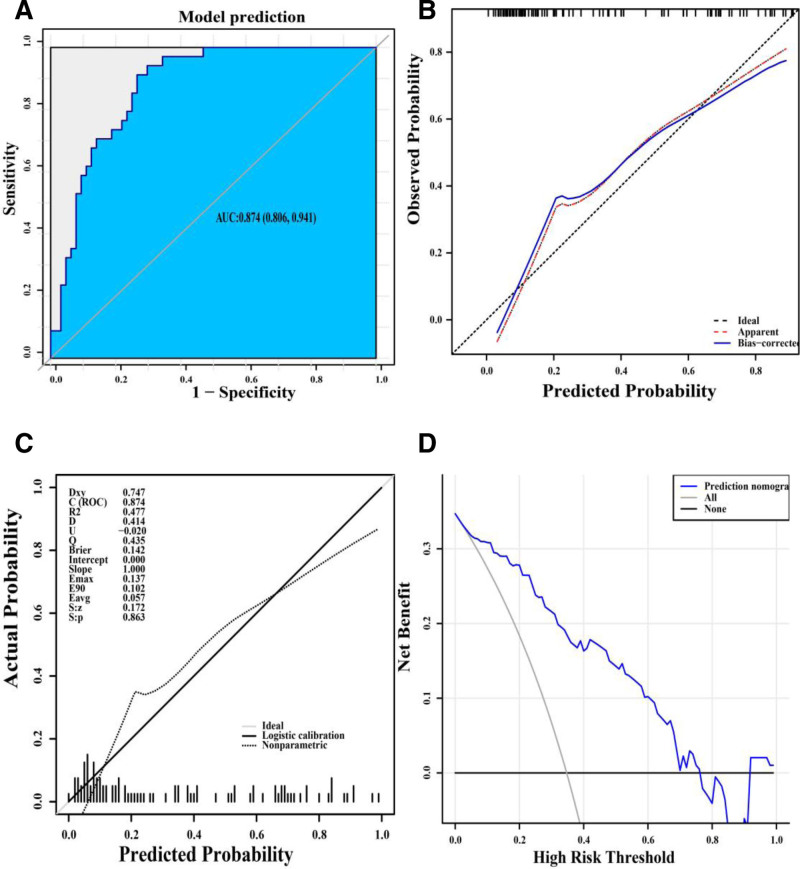
The discrimination and calibration assessment of the model. (A) Receiver operating characteristic curves for the nomogram. (B) Calibration curve for the nomogram to predict the probability of in-hospital mortality with bootstrap sampling validation. The red dotted line represents the performance of the nomogram, whereas the blue solid line corrects for any bias in the nomogram. The dashed line represents the reference line where an ideal nomogram would lie. (C) Calibration curve for the nomogram to predict the probability of in-hospital mortality with leave-one-out cross-validation. (D) Decision analysis curves showing the clinical usefulness of the nomogram prediction model. The abscissa represents threshold probability, the ordinate represents net benefit for patients. The horizontal line represents no clinical benefit for all patients without prediction and intervention. The gray line represents the clinical benefit of intervention for all patients, and the blue curve represents the clinical benefit of using the nomogram prediction model.

The nomogram was assessed using the AUC, concordance index, and calibration curve. As shown in Figure 4A and Table 2, the AUC for in-hospital mortality was 0.874 (95% confidence interval: 0.806–0.941) with a negative predictive value of 94%, a positive predictive value of 64.58%, a Youden’s index of 1.646 respectively. Figure 4B demonstrates that 200 bootstrap sampling validation was used to evaluate model accuracy, yielding a corrected concordance index of 0.846 and a calibration slope of 1.0. In Figure 4C, we used 8-fold leave-one-out cross-validation to assess the model, achieving a corrected concordance index of 0.849 and a calibration slope of 1.0. Table 3 indicates the model’s calibration was satisfactory, supported by the Hosmer–Lemeshow test (χ^2^ = 8.125, *P* = .421) and a brier score of 0.142 (mean 0.167 in cross-validation).

**Table 2:** The receiver operating characteristic curve analysis of model to predict prognosis of patients with acute diquat poisoning.

	AUC	95% CI	Youden’s index	Negative predictive value (%)	Positive predictive value (%)	Sensitivity (%)	Specificity (%)
Model	0.874	0.806–0.941	1.646	94	64.58	0.9118	0.7344

AUC = area under the curve, CI = confidence interval.

**Table 3:** The predictive performance of the final prediction model based on 8-fold cross-validation.

Model performance	AUC	95% CI	Brier score	Hosmer–Lemeshow test χ^2^	*P* value
Original performance	0.874	0.806–0.941	0.142	8.125	.421
Internal cross-validation	Mean 0.849		Mean 0.167		

AUC = area under the curve, CI = confidence interval.

### 3.5. Decision analysis curve for the predictive nomogram

A decision analysis curve (see Fig. 4D) shows that the net benefits of the predictive nomogram were significantly higher than those of the 2 extreme cases (all patients either received no intervention or underwent an intervention) therefore, patients receive a net clinical benefit. This analysis showed that patients could benefit from the model when the threshold probabilities were 1% to 76%.

## 4. Discussion

This study developed and validated a prediction model for in-hospital mortality in acute diquat poisoning patients. The model, incorporating post-ingestion time, diquat plasma levels, plasma lactate concentration, occurrence of respiratory failure, and need for blood transfusion, showed excellent predictive accuracy. It serves as a valuable tool for early risk assessment and management in these patients. For example, a patient with acute diquat poisoning arrived at the hospital 100 minutes after ingestion, with a plasma diquat concentration of 1 mg/L and a plasma lactate concentration of 8 mmol/L. The patient experienced respiratory failure but did not receive a need for blood transfusion. According to the nomogram, 100 minutes post-ingestion corresponds to 17.5 points, and a 1 mg/L diquat concentration equals 9 points. Additionally, a plasma lactate concentration of 8 mmol/L and respiratory failure each add 27.5 points, while no need for blood transfusion scores 25 points. The total score of 106.5 suggests a 60% mortality risk during hospitalization.

Our 5-variable model effectively predicts in-hospital mortality for acute diquat poisoning, consistent with other studies. Meng et al identified initial plasma diquat levels and the 24-hour poisoning severity score as independent mortality indicators.^[19]^ Our prior research had also demonstrated that the plasma diquat concentration, along with the severity-index derived from plasma diquat concentration and post-ingestion time, were correlated with in-hospital mortality.^[20]^ This model corroborates these findings, as plasma diquat concentration and post-ingestion time were among the strongest predictors of mortality in our cohort. The inclusion of these parameters in our model highlights their critical role in the prognosis of acute diquat poisoning. plasma lactate concentration, a byproduct of anaerobic metabolism, is crucial for monitoring circulatory resuscitation in critically ill patients and correlates with disease severity.^[25,26]^ Zhu et al identified it as a key prognostic indicator in diquat poisoning cases, underscoring its importance in early severity assessment.^[16]^ Consistent findings across studies highlight the importance of plasma lactate concentration in assessing diquat poisoning severity early on. Decisions on need for blood transfusions and managing respiratory failure typically indicate the patient’s hematologic and respiratory status. Our model reveals that both factors significantly impact in-hospital mortality risk, underscoring the need for early organ function evaluation in assessing diquat poisoning severity.

Previous studies indicated a 25% to 60% mortality rate for acute diquat poisoning.^[17–19]^ This study found an in-hospital mortality rate of 34.7%, aligning with past research. Aggressive treatments like blood purification, extracorporeal membrane oxygenation, lung transplantation, and multidisciplinary management might improve patient outcomes.^[14,27]^ But further randomized controlled trials or large-scale cohort studies are still needed to confirm. Lv et al developed a nomogram using clinical data to enhance risk prediction and treatment decisions for acute diquat poisoning patients.^[17]^ Their study found an average post-ingestion time of 7.50 hours, while ours reported 4.5 hours. In acute diquat poisoning, even a 1-hour difference can significantly impact patient outcomes. Thus, our research is better for early prognostic predictions and highlights the need for proactive measures during the initial stages to improve outcomes.

### 4.1. Future directions

Future research should focus on conducting large-scale, multicenter clinical trials and prospective validation of the model in diverse healthcare settings. Additionally, exploring the integration of novel biomarkers, such as inflammatory markers or genetic profiles, and advanced imaging techniques could further enhance the model’s predictive capabilities. Investigating the impact of early intervention strategies guided by the model’s risk stratification on patient outcomes would also provide valuable insights.

### 4.2. Clinical implications

The prediction model established in this study holds significant clinical importance. Its development could offer valuable insights for future intervention studies. Additionally, the model can provide some information for discussing prognosis with patients and their families.

### 4.3. Strengths and limitations

Our model offers several advantages: it uses easily obtainable baseline characteristics, demonstrates good discrimination and accuracy (as shown by AUC and calibration), and performs well in 200 bootstrap sampling validation and leave-one-out cross-validation. We developed an accurate predictive model for in-hospital mortality in acute diquat poisoning, aiming to help clinicians improve acute diquat patient outcomes.

This study has several limitations: Firstly, it is observational and cannot confirm causation. Secondly, it was conducted in a single hospital without internal split-sample validation and external validation, possibly limiting its regional applicability. Thirdly, biomarkers such as procalcitonin and neutrophil gelatinase-associated lipocalin, while discussed in prognostic literature,^[15,16]^ were not routinely measured in our cohort (missing data >50%) and thus excluded. Future studies should standardize biomarker collection to validate their utility.

## 5. Conclusion

In conclusion, our study presents a validated prediction model for in-hospital mortality among patients with acute diquat poisoning. The model’s strong performance underscores its potential utility in clinical practice, offering a valuable tool for early risk assessment and management. Continued research and external validation will be essential to ensure its widespread applicability and to explore its role in improving patient outcomes.

## Acknowledgments

We also thank Jie Liu, PhD (Chinese PLA General Hospital), and Jinbao Ma (Xi’an Chest Hospital) for their feedback.

## Author contributions

**Data curation:** Xian Chen, Haike Du, Xiaoming Jiang.

**Software:** Min Zhao.

**Writing – original draft:** Ye Zhang.

**Writing – review & editing:** Yingmin Ma.
